# Adverse Drug Events in Older Hospitalized Patients: Results and Reliability of a Comprehensive and Structured Identification Strategy

**DOI:** 10.1371/journal.pone.0071045

**Published:** 2013-08-05

**Authors:** Joanna E. Klopotowska, Peter C. Wierenga, Clementine C. M. Stuijt, Lambertus Arisz, Marcel G. W. Dijkgraaf, Paul F. M. Kuks, Henk Asscheman, Sophia E. de Rooij, Loraine Lie-A-Huen, Susanne M. Smorenburg, on behalf of the WINGS Study Group

**Affiliations:** 1 Department of Hospital Pharmacy, Academic Medical Center, Amsterdam, the Netherlands; 2 Department of Hospital Pharmacy, Deventer Hospital, Deventer, the Netherlands; 3 Department of Internal Medicine, Academic Medical Center, Amsterdam, the Netherlands; 4 Clinical Research Unit, Academic Medical Center, Amsterdam, the Netherlands; 5 Consultant internal medicine, HAJAP, Amsterdam, the Netherlands; 6 Department of Internal Medicine and Geriatrics, Academic Medical Center, Amsterdam, the Netherlands; Charité University Medicine Berlin, Germany

## Abstract

**Background:**

Older patients are at high risk for experiencing Adverse Drug Events (ADEs) during hospitalization. To be able to reduce ADEs in these vulnerable patients, hospitals first need to measure the occurrence of ADEs, especially those that are preventable. However, data on preventable ADEs (pADEs) occurring during hospitalization in older patients are scarce, and no ‘gold standard’ for the identification of ADEs exists.

**Methodology:**

The study was conducted in three hospitals in the Netherlands in 2007. ADEs were retrospectively identified by a team of experts using a comprehensive and structured patient chart review (PCR) combined with a trigger-tool as an aid. This ADE identification strategy was applied to a cohort of 250 older hospitalized patients. To estimate the intra- and inter-rater reliabilities, Cohen’s kappa values were calculated.

**Principal Findings:**

In total, 118 ADEs were detected which occurred in 62 patients. This ADE yield was 1.1 to 2.7 times higher in comparison to other ADE studies in older hospitalized patients. Of the 118 ADEs, 83 (70.3%) were pADEs; 51 pADEs (43.2% of all ADEs identified) caused serious patient harm. Patient harm caused by ADEs resulted in various events. The overall intra-rater agreement of the developed strategy was substantial (κ = 0.74); the overall inter-rater agreement was only fair (κ = 0.24).

**Conclusions/Significance:**

The ADE identification strategy provided a detailed insight into the scope of ADEs occurring in older hospitalized patients, and showed that the majority of (serious) ADEs can be prevented. Several strategy related aspects, as well as setting/study specific aspects, may have contributed to the results gained. These aspects should be considered whenever ADE measurements need to be conducted. The results regarding pADEs can be used to design tailored interventions to effectively reduce harm caused by medication errors. Improvement of the inter-rater reliability of a PCR remains challenging.

## Introduction

Patient harm due to medication, also known as Adverse Drug Events (ADEs), is the second most frequent complication occurring during hospitalization [1]–[4]. Between 6 and 30% of hospitalized patients experience an ADE during their hospitalization [5]. A recent Dutch study estimated that on average, an ADE can result in an excess length of stay of 6.2 days and additional costs of €2,507 [6]. Older patients are at higher risk for ADEs [7]. This higher risk is often related to the presence of multimorbidity and related polypharmacy [8], as well as age-related changes in pharmacokinetics and pharmacodynamics that influence drug elimination and response [9].

Because of ageing and an increasing life expectancy [10], the reduction of ADEs in these vulnerable patients has become a major patient safety goal in various settings [11], [12].

Furthermore, as stated by the Expert Group on Safe Medication Practices, proper steps should be taken to establish appropriate methods to identify ADEs, with the aim of evaluating the effect of medication safety practices and initiatives intended to reduce ADEs [13]. Preventable ADEs (pADEs) should be specifically addressed, i.e., harm caused by medication errors [14], [15]. However, data on pADEs occurring in older patients during hospitalization are scarce [1], [16]–[19]. Furthermore, the type of ADE identification method used can have consequences for the insight gained into the occurrence of ADEs in a specific setting. The lesser that is known about the extent of local medication safety risks, the more comprehensive the ADE identification method used should be [20].

A patient chart review (PCR) has been a widely used method because of its high ADE yield and its specificity in detecting pADEs [20]–[27]. This method can be used prospectively or retrospectively. Usually, nurses, physicians, or pharmacists review hospital data such as medical and nursing notes, medication charts, and laboratory results to search for events that could have been caused by medication. When identified, such events are assessed, usually by physicians and/or pharmacists, for the drug causality, severity, and preventability, and this assessment takes into account all available and relevant information on the considered case [28], [29]. Especially when assessing complex cases, such as those of older patients, the use of clinical judgment is mandatory. However, PCR is time-consuming, ADEs can be easily missed because of the often unstructured review process, and its implicit character often results in low inter-rater reliability [21], [30], [31].

To circumvent these limitations, use of explicit screening tools is advocated [20], [30], [32]. The Institute for Healthcare Improvement (IHI) developed an explicit screening tool specifically for the detection of ADEs in hospitalized patients [33]. This so called ‘trigger-tool’ is a list of sentinel words, for example specific medications or abnormal laboratory values, that could indicate the occurrence of an ADE [23], [30]. If a trigger is found in a patient chart, this chart is then further reviewed by experts to assess whether an ADE has occurred. The use of this trigger-tool has been shown to improve the sensitivity as well as the reliability of ADE measurements [20], [30], [32]. However, the IHI ADE trigger-tool was only validated in the general inpatient population in the United States, and developed to measure the overall level of harm [30], [33]. A study by Franklin et al. [34] showed that when IHI ADE trigger-tool was applied in a British hospital, whole classes of pADEs were missed.

Therefore, when prompted with the need to conduct first-time measurement of ADE occurrence in older patients during hospitalization, we chose a PCR as the basis, structured it, and combined it with the IHI ADE trigger-tool [33] as an aid. Our aim was to gain a more comprehensive and reliable ADE measurement. In this paper, we present results obtained by this ADE identification strategy when applied in a cohort of 250 older hospitalized patients. Additionally, the intra-rater and inter-rater reliability were evaluated.

## Methods

### Ethics Statement

The integral WINGS study protocol [35] was presented to The Medical Ethics Committee of the University of Amsterdam. The Medical Ethics Committee discussed the protocol and exempted it from review and official approval. According to the Dutch Medical Research Involving Human Subjects Act (WMO), such review and approval was not required because the study did not involve direct interaction with human subjects. This research used retrospective data to assess the occurrence of ADEs. Therefore, the integrity of the patients was not influenced and all patient data were analyzed anonymously by coding every included patient with a 6-digit number.

### Participants and Setting

For the purpose of this study, 250 patients from the baseline measurement of ‘Ward-oriented pharmacy In Newly admitted Geriatric Seniors’ (WINGS) study were included [35]. The patients were 65 years and older, used five or more medications upon admission and were consecutively admitted to Internal Medicine wards of the Academic Medical Centre, Amsterdam (university teaching hospital with 1003 beds) for 24 hours or longer between April and November 2007. Patients were excluded if they were admitted for planned chemotherapy, radiation therapy or transplantation, or if they were transferred from other non-medical wards within the same hospital or other hospitals.

The WINGS study was conducted within the CAREFUL (pharmacist Coordinated ADE Reducing Efforts For Use in all Levels of healthcare) research program based on a cooperation of Leiden University Medical Centre, Academic Medical Centre Amsterdam, University Medical Centre Groningen and University Medical Centre Utrecht/Utrecht University, The Netherlands.

### ADE Identification Strategy

The overall criteria for the ADE identification strategy were as follows: 1) the strategy should have a high ADE yield in order to gain comprehensive insight into the occurrence and types of ADEs, 2) it should account for the causality, severity, and preventability of ADEs, and 3) it should reliably measure potential changes in ADE occurrence as a result of future medication safety interventions.

We addressed the concerns about the limitations of a PCR in four ways.

First, to assure a structured and reliable data collection and ADE assessment, we developed a Case Report Form (CRF) that was based on previous research on adverse events [36], drafted a manual for this CRF, provided training in data collection for nurses and pharmacy students involved in data collection in this study, and provided training in PCR and ADE assessment for expert team members.

Second, to assure comprehensive data collection [13], we incorporated the IHI ADE trigger-tool [33] into the CRF. However, we did not use this trigger-tool for patient pre-selection because of aforementioned limitations. In contrast, in the present study, patient charts in which triggers were detected, as well as patient charts in which no triggers were detected, underwent further review by the expert team. Also, several modifications to the IHI ADE trigger-tool [33] were necessary to account for drug formularies and laboratory tests references prevailing in the Netherlands (Table S1).

Third, no restrictions were made regarding medication process stages at which ADEs can occur, and broad and internationally accepted definitions were applied.

Fourth, we assigned a multidisciplinary team consisting of both a physician and a pharmacist to identify and assess ADEs because their knowledge and experience is complementary [37], [38]. Therefore, such an approach could increase the ADE yield of the ADE identification strategy.

#### CRF and data collection

The CRF included the following components:

general patient characteristics,
laboratory findings including therapeutic drug monitoring findings: a spreadsheet to chronologically register any decreased or increased laboratory values,
diagnostic procedures: a spreadsheet to chronologically register any diagnostic procedures such as gastroscopy, echocardiograms, microbiology findings, consultations by other medical specialists,
allergies,
reason(s) for hospital admission and when applicable in-hospital transfers to intensive care or medium care units,
medication (home, hospital, discharge, as needed medication),
symptoms/complaints upon admission and during hospitalization: a spreadsheet to chronologically register pre-defined symptoms/complaints such as bleeding, hypotension, constipation, skin rash,
(differential) diagnoses upon admission and during the hospital stay,
when applicable, information regarding readmission within three months of the reviewed hospitalization.

Using the manual, trained research nurses and pharmacy students gathered data about the index hospitalization of the included patients and completed CRFs based on these data. The triggers included in the modified IHI ADEs trigger-tool were checked off by the research nurses or pharmacy students when they were detected. As mentioned before, patient charts in which triggers were detected, as well as charts in which no triggers were detected, underwent further review by the independent expert team.

An additional information source about medication use were print-outs of drug safety alerts overridden by the physicians while prescribing. In all participating hospitals computerized physician order entries (CPOEs) were operational. All CPOEs used in Dutch hospitals use the Dutch drug database ‘G-standaard’, which contains safety information on all drugs registered in the Netherlands, including drug-drug interactions, duplicate orders and dosing guidelines. It also provides standardized alert texts, i.e., a Clinical Decision Support (CDS) [39], [40]. When physicians enter prescriptions, alerts are shown on screen intrusively. Alerts show the drugs involved, their dosage regimens, and an explanation including a recommendation. Overridden alerts are logged for hospital pharmacy review [40]. This review is usually conducted by a hospital pharmacist once daily. If an overridden drug safety alert is judged by a hospital pharmacist as clinically relevant, he or she contacts by telephone either a nurse or a physician to provide additional advice. This advice can either be accepted, or rejected based on personal clinical opinion. When changes in pharmacotherapy are required, usually the physician changes the drug orders in the CPOE.

#### Definitions

An *Adverse Drug Event (ADE)* was defined as any harmful event occurring during drug therapy and resulting either from appropriate care (*nonpreventable ADEs*) or from medication errors (*preventable ADEs*) [41]. ADEs resulting in clinical symptoms (with or without abnormal laboratory values), as well as ADEs resulting in abnormal laboratory values only, were included. In the present study we focus on ADEs occurring during hospitalization.

The *causality* between a drug and an adverse event was assessed using an adapted causality assessment of the World Health Organization – Uppsala Medical Centre (WHO-UMC) [42]. Adaptation of the WHO-UMC causality assessment was necessary to be able to also assess ADEs due to drug omissions, since only drug intake (i.e. drug commission) is assumed as the starting point. The following modifications were made: option ‘drug omission’ was added whenever ‘drug intake’ was stated, option ‘drug initiation’ was added whenever ‘drug withdrawal’ was stated, and rechallenge could be either initiating a drug previously withdrew or withdrawing a drug previously initiated.

The *severity* of ADEs was scored according to the Common Terminology Criteria for Adverse Events version 3.0 (CTCAEv3) developed by the U.S. National Cancer Institute, which includes the following 5-points scale of seriousness: mild, moderate, severe, life-threatening, and fatal [43]. In the present study no restrictions were made regarding the ADE severity.

An ADE was judged as *preventable* if caused by a medication error. *Medication errors* included both errors of commission or omission [41]. Medication errors were classified according to the Dutch Central Medication Incidents Registration [44] and were defined as errors in drug prescribing, dispensing, administration, or monitoring [44]. To assess whether a medication error occurred, we utilized prevailing national [45] and local pharmacotherapy guidelines.

#### ADE assessment

The independent expert team consisted of a senior physician specializing in internal medicine (LA) and a senior clinical pharmacist specializing in geriatric medicine (CS), both of whom are well-acquainted with the PCR methodology.

Both experts received all data gathered about the index hospitalization and used the completed CRFs to guide them through the chart review. First, the two experts reviewed all data independently and recorded what in their opinion could be ADEs. Subsequently, they presented their findings during scheduled meetings. Events discussed were either events identified only by the physician, or only by the pharmacist, or identified by both experts. For each event brought forward during scheduled meetings, the experts shared their knowledge on the specific case, and conducted a joined causality assessment according to adapted WHO-UMC criteria. In the present study, only events assessed as having possible, probable, or nearly certain causality with drug commission or omission were registered. These ADEs were further assessed by the experts on severity, preventability, and when applicable, on type of medication error. In cases where there was initial disagreement between the experts during joint ADE assessment, this disagreement was solved by a consensus between the experts.

**Figure 1:**
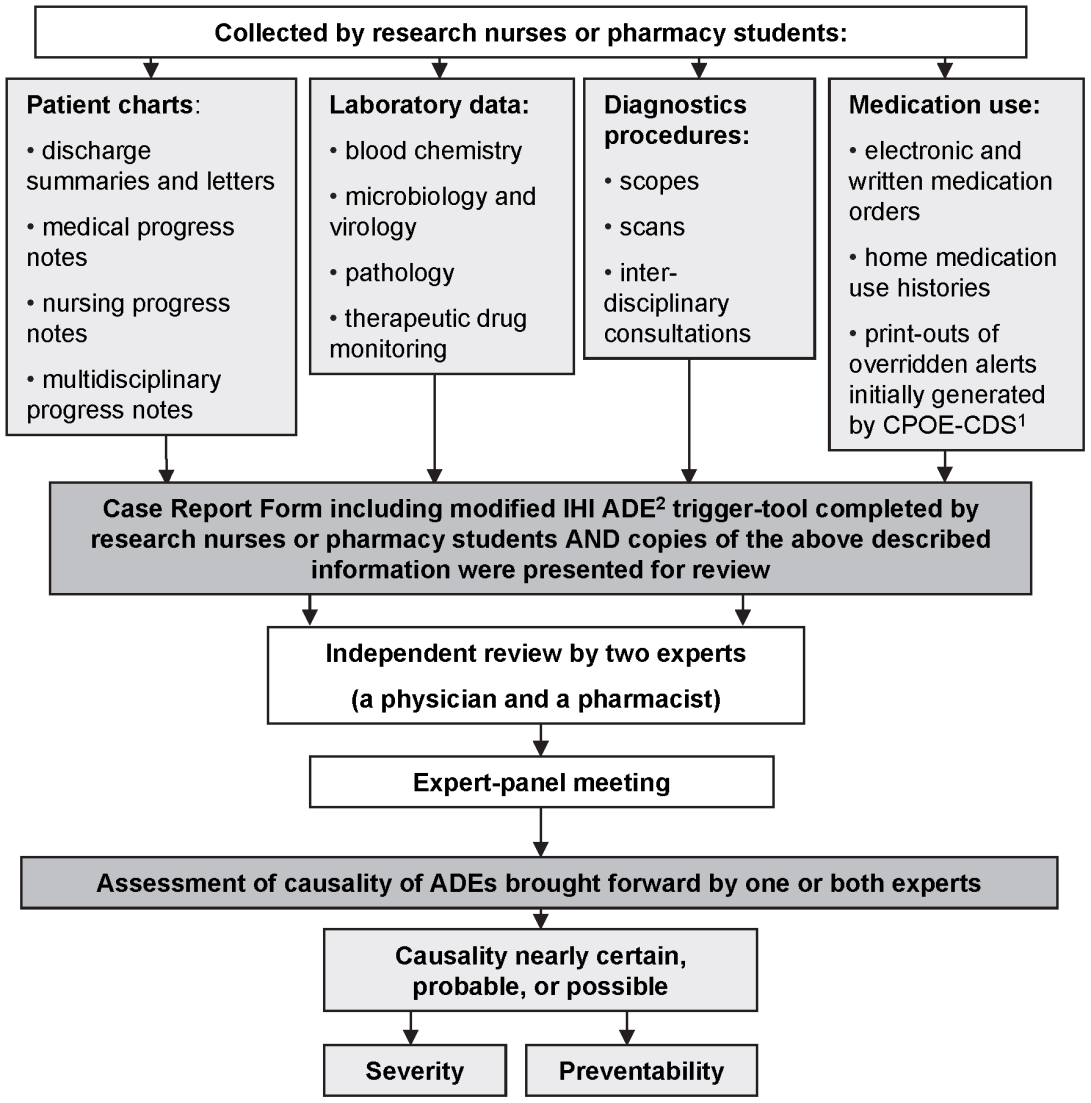
Flow-chart of the Adverse Drug Events identification and assessment processes. ^1^ CPOE-CDS - Computer Physician Order Entry with Clinical Decision Support. ^2^ ADEs - Adverse Drug Events.

A flowchart of ADE identification and assessment strategy is shown in Figure 1. No attempt was made to assess sensitivity or specificity of this strategy, as a clear “gold standard” for ADE identification is lacking [20], [22]–[27].

### Reliability Assessment

The reliability of the developed ADE identification strategy was assessed by calculating the intra- and inter-rater agreements for overall ADE occurrence, causality, severity, and preventability. The intra-rater agreement was defined as the test-retest of ADE judgments following a repeated ADE identification by the assigned expert team (LA and CS combined as a team compared at two different time points). For this purpose, a random sample of 10% of the included patients (25 cases) was re-reviewed by the assigned expert team one year after their first review in 2007. The inter-rater agreement was defined as the test-retest reliability of the ADE judgments following repeated ADE identification by a different physician-pharmacist expert team, which consisted of a senior physician specializing in internal medicine (HA) and a senior hospital pharmacist (PK), i.e., LA and CS combined as a team versus HA and PK combined as a team. Prior to the reviews, the second pair of experts received training in the method applied in the present study. Except for one accidental replacement, the same random sample of 10% of the included patients (25 cases), which was reviewed by CS and LA in 2007, was also reviewed by the second expert team (HA and PK).

### Statistical Methods

The chi-square test was used to compare the proportions of pADEs and npADEs. To estimate the intra- and inter-rater reliabilities for judgments on the occurrence of ADEs, we calculated Cohen’s kappa (κ; with 95% confidence interval) and the corresponding percentages of positive and negative agreement (single measure agreement). Furthermore, inter-rater and intra-rater agreements were also calculated for judgments on the causality, preventability, and severity of ADEs. A kappa value of 0.00 was considered to be poor agreement, 0.01–0.20 to be slight agreement, 0.21–0.40 to be fair agreement, 0.41–0.60 to be moderate agreement, 0.61–0.80 to be substantial agreement, and 0.81–1.00 was considered to be almost perfect agreement [46]. In the absence of a prior sample size calculation for this sub-study of the WINGS study [35], the results are reported at the descriptive level. A P-value smaller than 0.05 was considered statistically significant. All analyses were performed with SPSS version 18.0 (Chicago Illinois) and PEPI (Pairsetc) version 1.11 (Copyright J.H. Abramson 2003–4).

## Results

The mean age of the 250 included older hospitalized patients was 76.9 years (SD±7.5). Most patients were acutely admitted to the hospital (85.2%; Table 1). Upon admission, these patients were taking an average of 7.3 medications (SD±3.2), and during hospitalization, they were taking an average of 11.0 medications (SD±4.1).

**Table 1:** Characteristics of the included patients.

Characteristic	Patients
	(N = 250)
Mean age in years, ± SD	76.9±7.5
Female, n (%)	133 (53.2)
Living independent, n (%)	211 (84.4)
Acute admission, n (%)	213 (85.2)
Median days of hospitalization*, (interquartile range)	5.9 (3.6, 9.6)
Mean no. of medications used upon admission, ± SD	7.3±3.2
Mean no. of medications used during hospitalization, ± SD	11.0±4.1
Mean no. of chronic diseases, ± SD	3.2±1.7
Charlson Comorbidity Index score, n (%)	
0–2 points	132 (52.8)
3 points and higher	118 (47.2)
MDRD eGFR** (ml/min/1.73 m2), n (%)	(n = 240)
> = 60	94 (39.2)
30–59	89 (37.1)
<30	57 (23.7)

SD - standard deviation, MDRD eGFR - Modification of Diet in Renal Disease estimated Glomerular Filtration Rate.

* Days of hospitalization equals the length of stay on one of the internal medicine wards in days.

** MDRD eGFR; for ten patients no laboratory tests were obtained during hospitalization to assess renal function.

Using the ADE identification strategy, the expert team (LA and CS) identified a total of 118 ADEs, yielding a rate of 47.2 ADEs per 100 hospitalizations (Table 2). These 118 ADEs occurred in 62 patients; 83 ADEs (70.3%) were assessed as preventable, of which 51 (43.2% of all ADEs identified) caused serious patient harm (severe, life-threatening, or fatal patient harm). The 83 pADEs occurred in 37 patients. Overall, pADEs were more serious in comparison to npADEs (p = 0.001; Table 2). The majority of ADEs (92 ADEs; 78.0%) was assessed as having nearly certain causality. Only 43 ADEs (36.4%) of which 25 (58.1%) serious ADEs were related to a trigger included in the modified IHI ADE trigger-tool, while of the 75 ADEs not related to the triggers (i.e. those identified by chart review only), 35 ADEs (46.7%) were serious (p = 0.230). Of the 118 ADEs, 32 ADEs (27.1%) were solely identified by the physician (LA), 28 ADEs (23.7%) solely by the pharmacist (CS), and 58 ADEs (49.2%) were identified by both the physician and the pharmacist.

**Table 2:** Characteristics of nonpreventable and preventable Adverse Drug Events identified during hospitalization.

Characteristic	Subcategory	NonpreventableADEs, no. (%)	Preventable ADEs, no. (%)	p-Value
		n = 35	n = 83	
Severity	Mild	15 (42.9)	10 (26.5)	p = 0.001
	Moderate	11 (31.4)	22 (41.0)	
	Severe	9 (25.7)	34 (41.0)	
	Life-threatening	0 (0.0)	15 (18.1)	
	Fatal	0 (0.0)	2 (2.4)	
Event type	Clinical symptoms	19 (54.3)	47 (56.6)	p = 0.815
	Abnormal laboratory values only	16 (45.7)	36 (43.4)	
Causality	Nearly certain (>90% certainty about causality)	28 (80.0)	64 (77.1)	p = 0.116
	Probable/likely (65–90% certainty about causality)	3 (8.6)	16 (19.3)	
	Possible (33–65% certainty about causality)	4 (11.4)	3 (3.6)	
Detected by	Modified IHI ADE trigger-tool	17 (48.6)	26 (31.3)	p = 0.075
	Chart review only	18 (51.4)	57 (68.7)	

ADEs - Adverse Drug Events; IHI - Institute for Healthcare Improvement.

Various types of ADEs were identified, and the patterns in frequencies differed between npADEs and pADEs (Table 3). Metabolic/laboratory events were the most common clinical manifestation related to ADEs (30.5%), followed by infections (11.0%), and coagulation events (10.2%). Of these most common ADE types, the majority (70.5%) was classified as pADEs. Table 4 shows that pADEs resulting in metabolic/laboratory events were mainly related to use of contra-indicated medications (10 of 24 pADEs), causing hypo- or hyperglycemia in case of blood glucose lowering drugs, or hypo- or hyperkalemia in case of diuretics or Renin-Agiotensin-Aldosterone-System (RAAS) inhibitors. Most pADE infection events (8 of 12 pADEs) were cases of inappropriate choice of empirical antibiotic therapy resulting in delayed recovery, or no clinical improvement of infections. The coagulation pADEs, were all raised INR events caused mainly (5 of 7 pADEs) by interaction between vitamin K antagonists and antibiotics/antifungal medication. Omissions of iron supplements in patients with chronic cardiovascular disease who were hospitalized due to (excessive) blood loss, in whom anemia was not (sufficiently) corrected, corresponded to 6 out of 7 blood/bone marrow pADEs. Omissions of laxatives in patients taking opiates causing constipation or ileus corresponded to 4 of 10 gastrointestinal pADEs.

**Table 3:** Type of events related to Adverse Drug Events during hospitalization.

Events classification*	All ADEs	npADEs	pADEs	Subgroups of events*
	no. (%)	no. (%)	no. (%)	(no. ADEs)
	n = 118	n = 35	n = 83	
Allergy/Immunology	3 (2.5)	1 (2.9)	2 (2.4)	Allergic reactions (3)
Auditory/Ear	1 (0.8)	1 (2.9)	0 (0.0)	Hearing loss (1)
Blood/Bone marrow	7 (5.9)	0 (0.0)	7 (8.4)	Anemia (6), Leukocytopenia (1)
Cardiac Arrhythmia	2 (1.7)	0 (0.0)	2 (2.4)	Bradycardia (1), AV-block 2^nd^ degree (1)
Cardiac General	7 (5.9)	0 (0.0)	7 (8.4)	Hyper- or hypotension (3), Cardiac infarction (2), Heart failure (2)
Coagulation	12 (10.2)	5 (14.3)	7 (8.4)	Raised INR (12)
Dermatology/Skin	3 (2.5)	2 (5.7)	1 (1.2)	Rash (2), Pruritis/Itching (1)
Endocrine	1 (0.8)	0 (0.0)	1 (1.2)	Thyroid function low (1)
Gastrointestinal	11(9.3)	1 (2.9)	10 (12.0)	Constipation (4), Nausea (2), Diarrhea (2), Ileus (1), Peptic ulcer (1), Bile reflux (1)
Hemorrhage/Bleeding	3 (2.5)	3 (8.6)	0 (0.0)	Hematoma (2), Gastrointestinal bleeding (1)
Hepatobiliary/Pancreas	2 (1.7)	0 (0.0)	2 (2.4)	Liver dysfunction/insufficiency (2)
Infection	13 (11.0)	1 (2.9)	12 (14.5)	Infections (13)
Metabolic/Laboratory	36 (30.5)	12 (34.3)	24 (28.9)	Hypo- or hyperglycemia (11), Hypo- or hyperkalemia (9), Raised LTs (9), Raised creatinine (5), Hypermagnesemia (1), Hypophosphatemia (1)
Musculoskeletal/Soft tissue	1 (0.8)	1 (2.9)	0 (0.0)	Muscle cramps (1)
Neurology	4 (3.4)	1 (2.9)	3 (3.6)	Somnolence (1), Somnolence causing a fall (1), Seizure (1), Confusion (1)
Pulmonary/Upper respiratory	2 (1.7)	0 (0.0)	2 (2.4)	Dyspnea (1), Respiratory distress (1)
Renal/Genitourinary	4 (3.4)	1 (2.9)	3 (3.6)	Acute kidney injury (3), Urinary retention (1)
Vascular	6 (5.1)	6 (17.1)	0 (0.0)	Phlebitis (6)

* Events and subgroups of events were classified according to Common Terminology Criteria for Adverse Events (CTCEA) version 3.0 [43].

ADEs - Adverse Drug Events; npADEs - nonpreventable Adverse Drug Events; pADEs - preventable Adverse Drug Events; AV – atrioventricular; INR - international normalization ratio; LTs – liverfunction tests.

**Table 4:** Type of medication errors resulting in preventable Adverse Drug Events during hospitalization.

Type of medication error	pADEs	Medication involved	Most frequent type of events
	no. (%)	(no. pADEs)*	(no. pADEs)
	n = 83		
**Prescribing error**	**71 (85.5)**		
Contra-indicated medication	20	Blood glucose lowering drugs, excl. insulins (5), Lipidmodifying agents (3), Diuretics (3), RAAS-inhibitors (2),Drugs for acid related disorders (2), Drugs forconstipation (1), Beta-blocking agents (1),Opioids (1), Other (2)	Metabolic/Laboratory (10), Gastrointestinal (2), Cardiac General/Arrhythmia (3)
Undertreatment	19	Iron preparations (6), Opioids (4), Antibacterialsfor systemic use (2), Diuretics (2),Antithrombotic agents (1), Corticosteroids for systemicuse (1), NSAIDs (1), Other (2)	Blood/Bone marrow (6), Gastrointestinal (5), Infection (2), Cardiac General (2)
Dosing errors	14	Antibacterials for systemic use (6), Psycholeptics (2),Cardiac therapy agents (2),Lipid modifying agents (1),Thyroid therapy agents (1), Other (2)	Metabolic/Laboratory (5), Neurology (3), Gastrointestinal (2), Cardiac General/Arrhythmia (2)
Inappropriate choice	9	Antibacterials for systemic use (8),Beta-blocking agents (1)	Infection (8), Cardiac General (1)
Drug-drug interactions	6	Antithrombotic agents (5),Antibacterials for systemic use (1)	Coagulation (5), Renal/Genitourinary (1)
Overtreatment	2	Diuretics (1), Mineral supplements (1)	Metabolic/Laboratory (1), Hepatobiliary/Pancreas (1)
Drug duplication	1	Blood glucose lowering drugs, excl. insulins (1)	Metabolic/Laboratory (1)
**Medication** **administration error**	**7 (8.4)**	Antibacterials for systemic use (3), Insulin andanalogues (2), Beta-blocking agents (1),Antithrombotic agents (1)	Metabolic/Laboratory (2), Infection (2)
**Monitoring error**	**5 (6.0)**	Antibacterials for systemic use (2), Diuretics (1),RAAS-inhibitors (1),Thyroid therapy agents (1)	Metabolic/Laboratory (4), Blood/Bone Marrow (1)

* Medication was classified according to Anatomical Therapeutic Chemical (ATC) classification by World Health Organization Collaborating Centre for Drug Statistics Methodology [73].

pADEs - preventable Adverse Drug Events; RAAS - Renin-Angiotensin-Aldosterone-System; NSAIDs - Non-Steroidal Anti-Inflammatory Drugs.

Prescribing contra-indicated medications, undertreatment, drug-drug interactions, and inappropriate choice are all sub categories of prescribing errors, the leading cause of pADEs in this study (85.5%). Preventable ADEs due to errors in administration or monitoring stage were rarely identified (Table 4). No pADEs due to medication errors in the dispensing stage were detected. Medication associated with the 83 pADEs was clustered in several therapeutic classes. The top five medication classes involved were: antibacterials for systemic use (22 pADEs, 26.5%), blood glucose lowering drugs including insulin (8 pADEs, 9.6%), diuretics (7 pADEs, 8.4%), antithrombotic agents (7 pADEs, 8.4%), and iron preparations (6 pADEs, 7.2%).

Diuretics, lipid modifying agents, beta-blocking agents, cardiac glycosides, RAAS-inhibitors, and antiadrenergic agents grouped as ‘drugs acting on cardiovascular system’ were together related to 20 pADEs (24.1%).

### Reliability Assessment

In nine out of the 25 patients who were randomly selected to measure the intra-rater reliability, the assigned expert team (LA and CS) identified no ADEs during their initial review in 2007. In the remaining 16 patients, 34 ADEs were identified (ten ADEs related to triggers and 24 ADEs detected by chart review only). During their second review, conducted one year later, the assigned expert team again identified all ten ADEs related to triggers from their initial review. However, two ADEs that were detected by chart review only were missed, and two additional ADEs that were detected by chart review only were identified. In Table 5, the calculated Cohen’s kappa values along with the percentages of positive and negative agreement are shown. The intra-rater agreement regarding the presence of an ADE was substantial (κ = 0.74). The intra-rater agreement regarding the causality, preventability and severity of the agreed-upon ADEs ranged from substantial to almost perfect (κ = 0.67 to κ = 0.93).

**Table 5:** The intra- and inter-rater reliability of the Adverse Drug Event identification strategy.

Process	Subcategory	Intra-rater reliability	Inter-rater reliability
		(95% CI)	(95% CI)
		% agreement: positive, negative*	% agreement: positive, negative*
Identification	Overall occurrence of ADEs (ADEs detectedby triggers and/or by the chart review only)	0.74 (0.50 to 0.98); 94, 80	0.24 (−0.05 to 0.53); 79, 44
Assessment	Causality of ADEs (nearly certain versusprobable/likely versus possible)*	0.67 (0.41 to 0.93); 84, 88, 97	0.19 (−0.11 to 0.49); 72, 69, 90
	Preventability of ADEs(medication errors yes or no)	0.68 (0.42 to 0.94); 86, 81	0.38 (0.04 to 0.71); 71, 67
	Severity of ADEs(mild or moderate versus serious)	0.93 (0.80 to 1.0); 96, 98	0.85 (0.66 to 1.0); 91, 94

ADEs - Adverse Drug Events.

* For the causality of ADEs, the agreement shown is an agreement per causality category: nearly certain, probable, or possible.

Table 5 also shows the inter-rater agreement results. In the set of 25 randomly selected patients used for the inter-rater measurement (24 patients from the set used for the intra-rater measurement in 2007 plus one new randomly selected patient), the assigned expert team (LA and CS) identified 37 ADEs (of which 10 ADEs were related to triggers) during their initial review, and the second pair of reviewers (HA and PK) identified 36 ADEs (of which 10 ADEs were related to triggers). The two teams of experts agreed upon 29 ADEs and disagreed upon 15 ADEs. Of the total of 11 ADEs that were related to triggers, the teams agreed upon nine. The inter-rater agreement on overall ADE occurrence, and preventability was fair (κ = 0.24 and κ = 0.38, respectively), and agreement was only slight for the causality (κ = 0.19). The inter-rater agreement for ADE severity was almost perfect (κ = 0.85).

## Discussion

By applying the ADE identification strategy to a cohort of older hospitalized patients, the assigned independent expert team was able to comprehensively identify various types of ADEs, of which the majority was preventable and/or resulted in serious patient harm. The strategy proved to be reliable if used by one team of experts.

In comparison to most ADE studies in older hospitalized patients [1], [17]–[19], [47]–[51], the ADE rate in the present study was 1.1 to 2.7 times higher. This high ADE yield seems to result not merely from a high number of mild and moderate ADEs. In contrast, of the 118 ADEs identified, 60 ADEs (50.8%) resulted in serious patient harm. We identified only one study, by Egger et al. [16], that reported a higher rate of ADEs during hospitalization, and their rate was 133.1 ADEs per 100 hospitalizations. The results gained by the ADE identification strategy may be explained by several methodological aspects, as well as specific aspects of the setting or the study itself.

### Methodological Aspects

In contrast to the present study, none of the studies cited above [1], [16]–[19], [47]–[51] used the IHI ADE trigger-tool [33] as an aid during PCR to enhance ADE identification. The trigger-tool methodology has been shown to increase the ADE yield merely because the review process is more structured, and the reviewers are more focused [32]. However, given that only 36.4% of ADEs identified in the present study were detected by the modified IHI ADE trigger-tool, a pre-selection of patients based on this tool would have substantially underestimated the occurrence of (serious) ADEs in our cohort of older hospitalized patients. In other words, the time invested in reviewing all included patients according to our strategy was worthwhile. This finding is in line with others [34] and raises concerns regarding the use of trigger-tools without prior validation in specific settings or in specific patient populations.

Up to now, several studies using computerized ADE surveillance have been published [52], [53]. However, specific for older inpatients, the use of such computerized tool as an aid for ADE identification was only reported by Egger et al. [16]. In this study, a computerized drug database was used along with PCRs by an expert team. This database automatically detected possible drug-drug interactions and drug side effects. In total, 64 out of 163 ADEs were identified by database signals, of which the majority (45 ADEs, 70.3%) were drug side effects signals. In contrast, in the present study the Dutch drug safety database [39] was incorporated in CPOEs and not used as a separate ADE surveillance tool. Also, it did not generate alerts related to drug side effects. These differences may explain the higher ADE yield in the study by Egger et al. [16] in comparison to the present study; database validation results were not provided by the authors.

The prospective data collection applied by Egger et al. [16] may have additionally contributed to their results. During prospective data collection, the patients included in a study are intensively monitored by researchers for ADEs occurring during hospitalization. Nurses and physicians working on the wards can be approached to clarify or report events and/or to present additional information [54].

Another explanation for the high ADE yield in the present study may be the assignment of both a physician and a pharmacist as expert reviewers. These two experts reviewed all patient charts to identify ADEs and assessed all identified ADEs for the causality, severity, and preventability. Especially in the often clinically complex cases of older patients, a physician reviewer is mandatory. However, the pharmacotherapy and medication safety knowledge of a hospital/clinical pharmacist is also of essential and added value. Phansalkar et al. [37] showed that pharmacist reviewers identified almost twice as many ADEs as non-pharmacists (p = 0.003). Only Egger et al. [16] appointed, similar to the present study, a multidisciplinary team consisting of physicians and a pharmacist to conduct PCRs and to assess ADEs.

In the present study, all types of medication errors, including omission of medications (i.e. undertreatment), were considered. The latter category was not taken into account by other ADE studies in older hospitalized patients [1], [16]–[19], [47]–[51]. Yet, undertreatment is common in older patients [55], and in the present study, 22.9% of pADEs were related to this type of prescribing error. In line with ADE studies in older [1], [16]–[19], [47]–[51] and other patient populations [14], [56], [57], the findings of the present study show that most pADEs (85.5%) were a result of medication errors occurring at the stage of prescribing. This could be expected, as a PCR is especially suitable for detecting prescribing errors and is less suitable for detecting medication errors in other medication processes [58]. However, prescribing errors are the most clinically relevant medication errors because they more often result in patient harm in comparison to errors in other medication processes [14].

### Specific Aspects of the Setting/Study

Prescribing contra-indicated medications, undertreatment, and dosing errors were the most frequent prescribing errors in the present study. These errors occurred despite CPOEs with CDS (CPOEs-CDS) operating in the participating hospitals. CPOEs-CDS have shown to be effective tools in reducing pADEs [59]. However, to capture the above-stated prescribing errors, CPOEs-CDS should combine data on diagnosis, medication use/underuse, and laboratory findings, and should provide specific dosing advices for older patients. Such advanced systems are not yet fully developed [59], [60]. On the other hand, having CPOEs-CDS in place in the participating hospitals may explain why only 9.9% of pADEs occurring at the stage of prescribing was a result of drug-drug interactions and duplicate medication orders errors. The fact that this type of pADEs still occurred is probably related to frequent overriding of the alerts by the physicians or to alert fatigue due to low specificity of the alerts [61]. The review of overridden alerts by hospital pharmacists seems also a weak barrier.

Nevertheless, even if advanced CPOEs-CDS will be available in near future, to improve prescribing in older patients, clinical judgment and expertise of medical professionals skilled in geriatric care and pharmacotherapy is irreplaceable. When prescribing for older patients, one should take into account life expectancy and quality of life, select essential medications, and avoid drugs with a poorer benefit-to-risk ratio [62]. Given the limited data on treatment effects in older patients, selecting appropriate medications in this patient population is even more challenging. Furthermore, multimorbidity, polypharmacy, and reduced physical and cognitive functions are other factors that need to be taken into account [11]. Therefore, because of this complexity of older patients’ cases, a multidisciplinary approach to care for older hospitalized patients, such as participation of a clinical pharmacist in medical/geriatric teams [63]–[66], seems appropriate.

However, at the time of this study, routine on-ward participation of clinical pharmacists was not offered by the hospital pharmacy departments of the participating hospitals. The clinical services were mainly reactive and included: on-call availability of hospital pharmacists for pharmacotherapy or toxicology consultations, review of overridden CPOEs-CDS alerts and consultations if needed, and therapeutic drug monitoring and advices. Clinical consultations by geriatricians were also available on request. This kind of organization of pharmacy and geriatric services is common in Dutch hospitals, as well as in hospitals in other countries [67], [68]. The fact that, in line with previous findings [1], [16]–[19], [47]–[51], the ADEs identified in the present study affected almost every organ system and involved various medication classes, emphasizes the need to rethink the organization of (hospital) care for older patients [69].

Furthermore, in the participating hospitals, the daily care of patients was provided by junior medical residents who were supervised by attending senior physicians. Gaps in geriatric knowledge and skills across all care settings and levels of medical experience are of major concern [68]. Recently, criteria to detect undertreatment and inappropriate medication use (e.g. contra-indicated medications) have shown promising results [55]. Because pADEs caused by these two types of medication errors were frequent in the present study, the use of these criteria by physicians while prescribing could be of additional value.

### Evaluation of the Reliability

The intra-rater agreement on all aspects of ADE assessment was substantial to almost perfect. Structuring the chart review and ADE assessment processes, providing a CRF, a manual, and trainings, may all have contributed to this result. However, despite these efforts, the inter-rater agreements for the occurrence of ADEs and ADE preventability remained fair (κ = 0.24 and κ = 0.38, respectively) and were only slight for the causality (κ = 0.19). Because both teams included senior pharmacists and senior internal medicine physicians, these findings indicate a high degree of subjectivity in the review process, rather than disparities in the experience level of the reviewers. The subjectivity might have been reduced by a discussion between all four reviewers and by sharing knowledge during expert panel meetings. However, such an approach has been shown to improve agreement within a pair of reviewers but not between pairs of reviewers [70]. This seems to be the case in the present study. In contrast, the inter-rater agreement for the severity was almost perfect (κ = 0.85). To assess severity, the CTCEA classification for adverse events were used [43]. The explicit character of this tool, which provides five categories of severity for various types of events, may have decreased the subjectivity of expert judgments, leading to more reliable and consistent results between the two expert teams involved. The use of explicit measures has been advocated in patient and medication safety studies to improve the understanding of the results and to facilitate the comparison between different settings and/or patient populations [20]. However, in the case of complex patients, a patient-specific approach requiring implicit judgments on the causality and preventability of ADEs is unavoidable.

The ADEs related to triggers, which were included in the intra-rater agreement measurement, were all identified during repeated review by our expert team (LA and CS), and nine out of 11 ADEs related to triggers, previously detected by our expert team, were also identified by the second team of experts (HA and PK). However, because only 36% of ADEs indentified in this study were related to triggers included in the modified IHI ADE trigger-tool, the advantages of this explicit approach on the inter-rater agreement remains limited.

### Limitations

This study has several limitations. Clearly, the ADE identification strategy would be too resource- and time-intensive to undertake routinely in the inpatient setting. However, as shown by the results of the present study such investment was worthwhile and should be considered whenever an initial and a detailed insight into ADE occurrence is required. Such insight is necessary to be able to develop tailored interventions for a specific setting. A tailored approach has shown to be successful [71].

Prospective data collection has been stated as preferred study type, especially for the identification of preventable events [54]. Mainly because medical teams working on the wards can be involved in the identification of events and provide additional information whenever necessary. When data are collected retrospectively, which was the case in the present study, documentation bias can occur because reviewers must relay on information recorded in patient charts to identify and assess ADEs [72]. On the other hand, collecting data prospectively can introduce bias due to higher awareness of physicians and nurses working on the wards [20]. Therefore, if the goal of an ADE measurement is to investigate an effect of interventions (purpose of the WINGS study [35]), retrospective data collection seems more appropriate [54]. Furthermore, the comprehensiveness of patient data available in the present study may have reduced the difficulty in assessing occurrence and preventability of ADEs by our experts.

Given the fair inter-rater reliability of the ADE identification strategy, generalization of present study results to other settings or patient populations should be exercised with caution. Furthermore, in the present study, the modified IHI ADE trigger-tool was not used independently of the PCR. Therefore, it is not possible to assess the individual contributions of this tool to the total ADE yield. Combining methods that have consistently identified ADEs, such as PCR and (computerized) trigger-tools is, however, advocated [58].

### Conclusions

By using structured and comprehensive PCR combined with a modified IHI ADE trigger tool as an aid, we were able to gain detailed insight into the types of ADEs occurring in older patients. This insight can be used to develop tailored interventions to effectively reduce pADEs. Several aspects of the ADE identification strategy may have contributed to the high ADE yield gained. Others can use this knowledge to make well-founded choices regarding methodology whenever ADEs need to be measured. The improvement of the inter-rater reliability of a PCR conducted by experts remains challenging. To be able to use the ADE identification strategy for evaluation of future interventions, the same team of experts must be assigned for ADE identification and assessment. Intensified training of the assessors within an expert team should be aimed for.

## Supporting Information

Table S1 **The Institute for Healthcare Improvement (IHI) Adverse Drug Event Trigger Tool as modified to the Dutch hospital setting.** *Antiemetics: alizapride, domperidone, droperidol, metoclopramide, prochlorperazine, ondansetron, granisetron, palonosetron, tropisetron, aprepitant, fosaprepitant **Anti-diarrheal drugs: loperamide, activated charcoal (mostly used in drug intoxication).
(DOCX)
