# Supplementary material for: Adverse Drug Events in Older Hospitalized Patients: Results and Reliability of a Comprehensive and Structured Identification Strategy
Source: PLoS One. 2013 Aug 5;8(8):e71045. doi: 10.1371/journal.pone.0071045 (PMC3733642; doi:10.1371/journal.pone.0071045)
Supplement: Table S1 — The Institute for Healthcare Improvement (IHI) Adverse Drug Event Trigger Tool as modified to the Dutch hospital setting. *Antiemetics: alizapride, domperidone, droperidol, metoclopramide, prochlorperazine, ondansetron, granisetron, palonosetron, tropisetron, aprepitant, fosaprepitant **Anti-diarrheal drugs: loperamide, activated charcoal (mostly used in drug intoxication). (DOCX) [file pone.0071045.s001.docx]

| **Trigger** | **Adapted Trigger** | **Potential problem identified** |
| --- | --- | --- |
| T1 | Clemastine/prednisone | Hypersensitivity reaction to a medication |
| T2 | Vitamin K (phytomenadione) | Overanticoagulation with vitamin K antagonist |
| T3 | Flumazenil | Oversedation with benzodiazepine |
| T4 | Antiemetics^*^ | Nausea/emesis related to medication use |
| T5 | Naloxone | Oversedation with narcotic |
| T6 | Anti-diarrheal drugs^**^ | Adverse Drug Events |
| T7 | Sodium Polystyrene Sulfonate rectal enema | Hyperkalemia related to medication effect |
| T8 | Activated partial thromboplastin time > 30 seconds | Overanticoagulation with heparin |
| T9 | International Normalization Ration > 6 | Overanticoagulation with vitamin K antagonist |
| T10 | White blood cell count < 3.5 x 10^9^ | Neutropenia related to medication use |
| T11 | Serum glucose < 2.8 mmol/L | Hypogylcemia related to insulin or oral anti-diabetic medication |
| T12 | Raising serum creatinine, 2.5 times the baseline | Renal insufficiency related to medication |
| T13 | *Clostridium difficile* positive stool | Exposure to antibiotics |
| T14 | Digoxin level > 2 µg/L | Digoxin toxicity |
| T15 | Lidocaine level > 6 µg/L | Lidocaine toxicity |
| T16 | Gentamicin/tobramycin levels peak > 6 g/L, through > 1 mg/L | Gentamicin/tobramycin toxicity |
| T17 | Amikacin levels peak > 50 mg/L, through > 4 mg/L | Amikacin toxicity |
| T18 | Vancomycin through level > 15 ml/L | Vancomycin toxicity |
| T19 | Theophylline level > 20 mg/L | Theophylline toxicity |
| T20 | Oversedation, lethargy, falls, hypotension | Related to overuse of |
| T21 | Rash | Medication related/Adverse Drug Event |
| T22 | Abrupt medication stop | Adverse Drug Event |
| T23 | Transfer to a higher level of care | Adverse Event |
